# Glucose-regulatory hormones and growth in very preterm infants fed fortified human milk

**DOI:** 10.1038/s41390-024-03166-8

**Published:** 2024-04-05

**Authors:** Kristine Holgersen, Martin Bo Rasmussen, Itay Zamir, Lise Aunsholt, Gitte Zachariassen, Per Torp Sangild

**Affiliations:** 1 Hans Christian Andersen Children’s Hospital, Odense University Hospital, Odense, Denmark; 2 Comparative Pediatrics and Nutrition, Department of Veterinary and Animal Sciences, University of Copenhagen, Copenhagen, Denmark; 3 Department of Clinical Sciences, Pediatrics unit, Umeå University, Umeå, Sweden; 4 Department of Neonatology, Rigshospitalet, Copenhagen, Denmark; 5 Department of Clinical Medicine, University of Copenhagen, Copenhagen, Denmark; 6 Department of Clinical Research, University of Southern Denmark, Odense, Denmark; 7 Open Patient data Explorative Network, Region of Southern Denmark, Odense, Denmark

## Abstract

**Background:**

Bovine colostrum (BC) contains a range of milk bioactive components, and it is unknown how human milk fortification with BC affects glucose-regulatory hormones in very preterm infants (VPIs). This study aimed to investigate the associations between hormone concentrations and fortification type, birth weight (appropriate/small for gestational age, AGA/SGA), milk intake, postnatal age, and body growth.

**Methods:**

225 VPIs were randomized to fortification with BC or conventional fortifier (CF). Plasma hormones were measured before, one and two weeks after start of fortification. ΔZ-scores from birth to 35 weeks postmenstrual age were calculated.

**Results:**

Compared with CF, infants fortified with BC had higher plasma GLP-1, GIP, glucagon, and leptin concentrations after start of fortification. Prior to fortification, leptin concentrations were negatively associated with growth, while IGF-1 concentrations associated positively with growth during fortification. In AGA infants, hormone concentrations generally increased after one week of fortification. Relative to AGA infants, SGA infants showed reduced IGF-1 and leptin concentrations.

**Conclusion:**

Fortification with BC increased the plasma concentrations of several glucose-regulatory hormones. Concentrations of IGF-1 were positively, and leptin negatively, associated with growth. Glucose-regulatory hormone levels were affected by birth weight, milk intake and postnatal age, but not closely associated with growth in VPIs.

**Impact:**

- Little is known about the variation in glucose-regulatory hormones in the early life of very preterm infants (VPIs).
- This study shows that the levels of glucose-regulatory hormones in plasma of VPIs are highly variable and modified by birth weight (appropriate or small for gestational age, AGA or SGA), the type of fortifier, enteral nutritional intake, and advancing postnatal age.
- The results confirm that IGF-1 levels are positively associated with early postnatal growth in VPIs, yet the levels of both IGF-1 and other glucose-regulatory hormones appeared to explain only a small part of the overall variation in growth rates.

## Introduction

Impaired postnatal growth is common in very preterm infants (VPIs, <32 weeks gestational age, GA) and associated with poor neurological development.^1–3^ It is therefore essential to secure optimal nutrition for these infants, which makes it relevant to add nutrient fortifiers to mother´s own milk (MOM) or donor human milk (DHM).^4,5^ A bovine colostrum (BC)-based fortifier has been suggested as an alternative to conventional fortifiers (CFs).^6^ BC is the first milk from cows produced after parturition. In vivo studies using preterm pigs as a clinically relevant model of preterm infants, have shown improved growth and gut maturation after BC feeding compared with DHM and/or formula feeding.^7–10^ The mechanisms of these BC effects are unknown, but the gut protective effects in preterm pigs remain even after pasteurization^11^ and may relate to high contents of intact bioactive milk proteins, compared with CFs. The latter are usually based on mature bovine milk (whey) protein and subjected to several industrial hydrolysis, heat treatment and filtration steps.^6,12–15^ The BC constituents include variable amounts of insulin and insulin-like growth factor-1 (IGF-1),^13–15^ anabolic peptides involved in pre- and postnatal growth, organ development and glucose metabolism.^16–22^ Even if not absorbed, such milk bioactives may have indirect effects on endocrine hormone production and body growth by improving gut growth, function and nutrient absorption.^23–26^

Preterm infants often show dysregulated insulin secretion and sensitivity, thereby inducing disturbances in glucose homeostasis^27–29^ that predispose these infants to growth deficits,^30^ early mortality and morbidities.^29,31,32^ Preterm infants also have increased risk of insulin resistance,^33^ metabolic syndrome and cardiovascular diseases^34–36^ later in life. In this regard, insulin action may interact with incretins (e.g. gastric inhibitory polypeptide, GIP, glucagon-like peptide-1, GLP-1) and other pancreatic peptides (e.g. C-peptide, glucagon, pancreatic polypeptide, PP).^37–40^ As yet, limited information is available about glucose-regulatory hormones in VPIs in response to nutritional input during the critical first weeks after preterm birth.

The objectives of this study were to investigate if plasma levels of glucose-regulatory hormones during the first weeks after very preterm birth, were affected by the type of nutrient fortifier, and if the hormone concentrations were associated with birth weight (appropriate/small for gestational age, AGA/SGA), enteral nutritional intake, postnatal age, and growth rates (ΔZ-scores from birth to 35 weeks postmenstrual age, PMA). Thus, glucose-regulatory hormones were measured in VPIs before the first fortified meal and approximately one and two weeks after the start of nutrient fortification with either BC or a CF product.

## Methods

### Study population and ethics

Data and samples were obtained as part of the FortiColos clinical trial, a previously published open-label randomized controlled multicentre trial, that compared nutrient fortification with BC or CF in infants born at GA 26 + 0 to 30 + 6 weeks between November 2017 and October 2020 in Denmark (Clinicaltrials.gov registration: NCT03537365).^41^ Infants included had enteral intakes >100 mL/kg/d at the start of fortification, a clinical condition requiring fortification according to local guidelines (e.g. blood urea nitrogen <5 mmol/L), no major congenital anomalies or gastrointestinal surgery, and had not received formula prior to enrollment. Participants were randomized using an online randomization program, REDCap^42^ in a server at the Region of Southern Denmark with a 1:1 allocation, random block sizes of 4–6, and stratified by SGA (defined as a birth weight Z score ≤−2 standard deviations (SD) for GA, according to ref. ^43^). The primary outcome in the original trial was to evaluate feasibility and safety of the alternative BC fortifier, aiming to achieve similar growth rates and morbidities (e.g. necrotizing enterocolitis, late-onset sepsis) between the BC and CF group. The participant flow chart is shown in Supplementary Fig. 1. Out of 453 eligible infants, 85 (19%) declined participation and 126 (28%) were not included due to other reasons (e.g. critically ill mother, parents did not speak Danish). Thus, 242 infants were randomized to receive either BC (*n* = 118) or CF (*n* = 124). Attending physicians withdrew seven infants from the study due to the clinical condition (one in the BC group, six in the CF group), and three infants were withdrawn from the study at the request of their caregivers (two in the BC group, one in the CF group).^41^ No blood samples were collected from seven infants (six in the BC group, one in the CF group), leaving 225 infants to be included in this study (109 in the BC group, 116 in the CF group). Due to variable and strict limitation of blood sample volumes (max 0.5 mL), total sample size varied for the different hormonal study endpoints. The study was approved by the ethical committee of the Region of Southern Denmark (S-20130010, S-20170095) and the Danish Data Protection Agency (17/33672). Written parental consent was obtained for all participants.

### Nutrition

A detailed description of the study protocol and fortification process, including detailed nutrient content and plasma amino acid concentrations two weeks after start of fortification, has been reported previously.^6,41^ Local nutritional guidelines were followed at each unit, considering the ESPHAN guidelines,^44^ introducing MOM as soon as possible after preterm birth, supplemented with DHM when needed. Fortification of MOM or DHM started when enteral intakes reached 100–140 mL/kg/d and blood urea nitrogen was <5 mmol/L (median postnatal day eight-nine).^41^ Infants were randomly assigned to receive human milk, containing either BC-based fortifier (ColoDan powder, Biofiber-Damino, Gesten, Denmark) or a bovine milk-based CF (PreNan FM85 powder, Nestlé, Switzerland), every two-three hours. Feeding volumes and amounts of fortification aimed to reach growth adhering to existing guidelines at each unit (i.e. not fixed by the protocol). The amount of fortification per milk volume was adjusted to provide equal amounts of total BC or CF protein per milk volume. Initially 1.0 g of fortification powder was added to 100 mL of human milk for both groups, increasing to a maximum of 2.8 g BC/100 mL and 4.0 g CF/100 mL within 3-4 of days (resulting in a maximum of +1.4 g protein/100 mL for both groups). The intervention continued until 34 + 6 weeks PMA.

### Data collection and analyses

Clinical (growth indices, morbidities, medication) and nutritional (fortification, enteral feeding volume of MOM, DHM) data were extracted from the FortiColos trial OPEN REDCap database. Glucose data were retrieved from the infants’ medical files and entered to the OPEN REDCap database. Preprandial blood samples were collected at three time points, before start of fortification (T0) and after 7 ± 3 (T1) and 14 ± 3 (T2) days of fortification. The vast majority of blood samples were collected just before the next meal, during the morning hours. Blood was collected in EDTA tubes, kept cooled on ice and centrifuged within one hour, without any additives. Plasma was frozen at −60 to −80 °C. IGF-1 concentrations at T0 (*n* = 197), T1 (*n* = 175) and T2 (*n* = 198) were determined using an enzyme-linked immunosorbent assay (ELISA, E-20, Mediagnost, Reutlingen, Germany). Intra- and inter-assay variability were 5.8% and 6.2%, respectively, as declared by the manufacturer. Active GLP-1, C-peptide, active GIP, glucagon, insulin, leptin and PP concentrations at T0 (*n* = 164), T1 (*n* = 195) and T2 (*n* = 196) were analyzed using an electrochemiluminescence assay (Multi-spot V-plex Metabolic panel 1, Meso Scale Diagnostics, Rockville, Maryland). Intra- and inter-assay variations were <7% for all analytes, as declared by the manufacturer. When concentrations were below the detection limit, the results were assigned to 50% of the limit of detection. Glucose concentrations at T0 (*n* = 112), T1 (*n* = 122) and T2 (*n* = 99) were analyzed using a blood gas analyzer (ABL 800, Radiometer, Broenshoej, Denmark). A homeostasis model assessment 2 (HOMA2) index was determined using established calculations (Diabetes Trials Unit, University of Oxford, http://www.dtu.ox.ac.uk/homacalculator/index.php, version 2.2.4, updated Nov 27, 2019). The following formula was used to calculate a quantitative insulin sensitivity check index (QUICKI: 1/(log (insulin μU/mL)+log(glucose mg/dL)). Infants were classified as receiving primarily MOM, DHM or ‘mixed human milk’ (MOM supplemented with DHM) based on the most frequently administered diet on the day of blood sampling. Total daily protein intake (g/kg/d) was calculated using extra protein from added fortifier and the following estimations of protein content in human milk; 1.7 g/100 mL for MOM,^45^ 0.9 g/100 mL for DHM,^46,47^ and 1.3 g/100 mL if the infant received mixed human milk. Growth outcome (early postnatal growth) was defined as ΔZ-scores for weight, length and head circumference (HC)^43^ from birth to end of intervention at 34 + 6 weeks PMA. Combining data from the two fortification groups, associations between ΔZ-scores and hormone concentrations, and age-related temporal changes in hormone concentrations in AGA and SGA infants, were assessed in infants with plasma samples available at postnatal age 7 ± 3 (T0), 14 ± 3 (T1) and 21 ± 3 (T2) days.

### Statistical analyses

Statistical analyses were performed using statistical software R (version 4.1.2, R Foundation for Statistical Computing, Vienna, Austria) or SAS (Version 9.4, SAS institute, Cary, North Carolina). Basic characteristics were compared between BC and CF infants with Student’s *t* test or Wilcoxon rank sum test for continuous variables and chi-square test for categorical variables. General linear models were used to compare hormone concentrations between the intervention groups. The model was tested for normality and homogeneity by plots, and data were log-transformed when required. The models were adjusted for SGA, GA at birth, and region (Eastern and Western Denmark), because clinical routines were known to vary between these regions, justifying adjustment for this possible confounder.^41^ Associations between hormone concentrations and daily nutritional intakes and ΔZ-scores for growth indices, as well as differences in hormone concentrations between AGA and SGA infants, were analyzed by general linear models, adjusted for intervention, GA at birth and SGA, as appropriate. Squared partial correlation coefficients for associations were adjusted likewise. Changes over time in hormone concentrations in AGA and SGA infants were analyzed using linear mixed-effects models adjusted for intervention and GA at birth, with infant ID as a random factor and post-hoc Tukey test to correct for multiple comparisons. *P* values < 0.05 were regarded as statistically significant.

## Results

### Bovine colostrum-based fortification and hormone concentrations

**Fig. 1:**
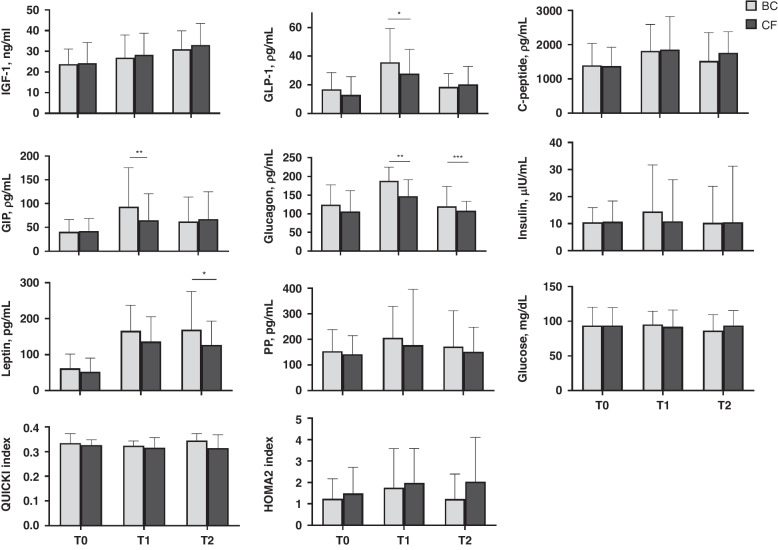
Fortification type (BC: bovine colostrum, CF: conventional fortifier) and glucose-regulatory hormones in very preterm infants. Plasma was sampled before start of fortification (T0), and after 7 ± 3 days (T1) or 14 ± 3 days (T2) of fortification and analyzed for IGF-1 (BC: *n* = 86–99, CF: *n* = 89–106), other hormones (BC: *n* = 74–98, CF: *n* = 90–104), glucose (BC: *n* = 52–61, CF: *n* = 47–60) and QUICKI/HOMA2 indices (BC: *n* = 38–53, CF: *n* = 43–58). The models were adjusted for gestational age at birth, small for gestational age and region. IGF-1 insulin-like growth factor-1, GLP-1 glucagon like peptide-1, GIP gastric inhibitory polypeptide, PP pancreatic polypeptide. Data is presented as median with interquartile range. *P < 0.05, **P < 0.01, ***P < 0.001.

Baseline characteristics of the entire cohort included in the intervention study are shown in Table 1. The intervention groups did not differ in their baseline characteristics and growth rate was unaffected by the intervention, as previously reported^41^ (Table 2). Differences in nutritional intake were observed with BC infants receiving a slightly higher enteral volume of human milk than CF infants at T1 and T2 (Table 2). The total daily amount of protein provided from fortification and milk intake, as well as the proportion of infants receiving primarily MOM, DHM or mixed human milk, were not significantly different between the groups at T1 and T2 (Table 2). Concentrations of IGF-1, GLP-1, C-peptide, GIP, glucagon, insulin, leptin, PP, and glucose before the first fortified meal (T0), and after one (T1) and two (T2) weeks of fortification for each intervention group are shown in Fig. 1, together with QUICKI and HOMA2 indices. At T0, no significant differences were found between the groups. At T1, GLP-1, GIP, and glucagon concentrations were ~20–30% higher in BC infants, with both glucagon and leptin concentrations being approximately 24% higher in this group at T2. No significant differences were noted in concentrations of IGF-1, C-peptide, insulin, PP, and glucose or in QUICKI and HOMA2 indices (Fig. 1). The results remain essentially the same in subanalysis including additional adjustment for baseline (T0) levels (except that leptin concentrations in BC infants were also higher at T1). After adjustment for daily enteral volume or total daily protein intake, C-peptide concentrations at T2 were significantly lower in the BC group, whereas leptin concentrations at T2 were no longer different between the groups. Also, after adjustment for total daily protein intake, IGF-1 concentrations at T1 were significantly lower in the BC group.

**Table 1:** Basic characteristics.

Characteristic	*n*	Entire study cohort	*n*	IGF-1 sub-cohort^a^	*n*	Other hormones sub-cohort^a^
*Perinatal variables*						
Bovine colostrum/Conventional fortifier, *n* (%)	225	109/116 (48.4/51.6)	101	54/47 (53.5/46.5)	94	48/46 (51.1/48.9)
Gestational age at birth, mean (SD), weeks + days	225	28 + 5 (1 + 3)	101	29 + 0 (1 + 2)	94	29 + 0 (1 + 2)
Males/females, *n* (%)	225	129/96 (57.3/42.7)	101	57/44 (56.4/43.6)	94	56/38 (59.6/40.4)
Multiple birth, *n* (%)	225	70 (31.1)	101	31 (30.7)	94	29 (30.9)
Small for gestational age, *n* (%)	225	52 (23.1)	101	31 (30.7)	94	18 (19.1)
Antenatal steroid treatment, *n* (%)	225	215 (95.6)	101	95 (94.1)	94	89 (94.7)
Chorioamnionitis, *n* (%)	222	8 (3.6)	101	2 (2.0)	94	3 (3.2)
Preeclampsia, *n* (%)	219	49 (22.4)	96	24 (23.8)	89	17 (19.1)
Abruptio placenta, *n* (%)	221	18 (8.1)	99	9 (9.1)	93	9 (9.7)
Prolonged premature rupture of membranes, *n* (%)	225	49 (21.8)	101	18 (17.8)	94	20 (21.3)
Cesarean section, *n* (%)	225	161 (71.6)	101	78 (77.2)	94	68 (72.3)
Apgar 5 min, mean (SD)	211	9.0 (1.7)	95	8.8 (1.9)	88	8.8 (2.0)
*Neonatal variables*						
Ventilator prior to intervention, *n* (%)	225	44 (19.6)	101	12 (11.9)	94	12 (12.8)
Treatment with systemic steroids, *n* (%)	225	10 (4.4)	101	4 (4.0)	94	3 (3.2)
Early-onset sepsis (5 days with antibiotics started within 2 days after birth), *n* (%)	225	25 (11.1)	101	8 (7.9)	94	5 (5.3)
Late-onset sepsis (5 days with antibiotics started 3 days after birth or later), *n* (%)	225	46 (20.4)	101	16 (15.8)	94	14 (14.9)
Intraventricular hemorrhage (IVH)						
No IVH, *n* (%)	222	186 (83.8)	100	88 (88.0)	93	80 (86.0)
Grade 1–2, *n* (%)	222	28 (12.6)	100	12 (12)	93	13 (14.0)
Grade 3–4, *n* (%)	222	8 (3.6)	100	0 (0)	93	0 (0)
Necrotizing enterocolitis, *n* (%)	222	7 (3.2)	100	3 (3.0)	93	2 (2.1)
Patent ductus arteriosus, *n* (%)	221	65 (29.4)	100	19 (19.0)	93	21 (22.6)
Periventricular leukomalacia (PVL), *n* (%)	218	3 (1.4)	99	0 (0)	92	0 (0)
Retinopathy of prematurity (ROP)						
No ROP, *n* (%)	214	196 (91.6)	97	90 (92.8)	90	83 (92.2)
Stage 1–2, *n* (%)	214	16 (7.5)	97	7 (7.2)	90	7 (7.8)
Stage 3–5, *n* (%)	214	2 (0.9)	97	0 (0)	90	0 (0)
Bronchopulmonary dysplasia (BPD), *n* (%)	219	39 (17.8)	98	15 (15.3)	91	13 (14.3)
*Anthropometry at birth*						
Weight, mean (SD), g	225	1168 (328)	101	1181 (319)	94	1223 (345)
Weight Z-score, mean (SD)	225	–1.12 (1.23)	101	–1.33 (1.19)	94	– 1.09 (1.24)
Length Z-score, mean (SD)	199	–1.36 (1.92)	92	–1.43 (1.94)	86	– 1.11 (1.86)
Head circumference Z-score, mean (SD)	201	–0.73 (1.0)	94	–0.82 (0.95)	86	– 0.65 (0.99)
*Anthropometry at end of intervention*						
Weight, mean (SD), g	223	2130 (361)	99	2071 (366)	92	2154 (349)
Weight Z-score, mean (SD)	223	–1.32 (1.08)	99	–1.50 (1.07)	92	–1.26 (1.06)
Length Z-score, mean (SD)	206	–1.77 (1.70)	92	–1.89 (1.78)	87	–1.68 (1.73)
Head circumference Z-score, mean (SD)	204	–0.66 (0.95)	89	–0.70 (0.93)	85	–0.56 (0.93)

^a^Includes only infants with plasma samples available at T0 (postnatal age 7 ± 3 days), T1 (postnatal age 14 ± 3 days) and T2 (postnatal age 21 ± 3 days).

**Table 2:** Baseline and enteral nutrition characteristics in very preterm infants receiving fortification with bovine colostrum (BC) or conventional fortifier (CF).

	*n*	BC	*n*	CF	*P*-value
*Perinatal variables*					
GA at birth, week + d, median	109	28 + 6 (26 + 0–30 + 6)	116	28 + 5 (26 + 0–30 + 6)	0.601
SGA, *n* (%)	109	26 (23.9)	116	26 (22.4)	0.798
Girls/boys, *n*/*n*	109	44/65	116	52/64	0.499
*Anthropometry*					
Weight at birth, g, mean ± SD	109	1172 ± 336	116	1164 ± 324	0.870
Weight at T1, g, mean ± SD	106	1265 ± 333	112	1297 ± 335	0.433
Weight at T2, g, mean ± SD	101	1438 ± 369	104	1501 ± 353	0.211
Weight, Z score, at birth, mean ± SD	109	−1.2 ± 1.2	116	−1.1 ± 1.2	0.629
Length, Z score, at birth, mean ± SD	97	−1.3 ± 2.0	102	−1.4 ± 1.8	0.696
HC, Z score, at birth, mean ± SD	97	−0.7 ± 1.0	104	−0.8 ± 1.0	0.567
Weight, Z score, at end of intervention, mean ± SD	107	−1.4 ± 1.1	116	−1.2 ± 1.1	0.119
Length, Z score, at end of intervention, mean ± SD	99	−1.9 ± 1.9	107	−1.6 ± 1.6	0.613
HC, Z score, at end of intervention, mean ± SD	97	−0.7 ± 1.1	107	−0.6 ± 0.8	0.548
Weight ΔZ-score from birth to end of intervention, mean ± SD	107	−0.3 ± 0.6	116	−0.1 ± 0.8	0.148
Length ΔZ-score from birth to end of intervention, mean ± SD	89	−0.4 ± 1.3	93	−0.3 ± 1.3	0.361
HC Δ Z-score from birth to end of intervention, mean ± SD	86	0.0 ± 0.8	95	0.1 ± 0.8	0.519
*Enteral nutrition*					
Volume of human milk given during intervention					
T1, mL/kg/day, median (min-max)	101	162 (135–224)	103	161 (109–251)	0.011
T2, mL/kg/day, median (min-max)	92	166 (99–235)	97	158 (0–209)	<0.001
Total protein provided from fortifier and human milk intake					
T1, g/kg/day, mean ± SD	95	4.2 ± 0.7	91	4.0 ± 0.8	0.084
T2, g/kg/day, mean ± SD	86	4.4 ± 0.8	86	4.1 ± 0.7	0.051
Primary milk type provided at T1					
MOM, *n* (%)	95	75 (78.9)	97	83 (85.6)	0.230
DHM, *n* (%)	95	11 (11.6)	97	8 (8.2)	0.440
Mixed milk, *n* (%)	95	9 (9.5)	97	6 (6.2)	0.396
Primary milk type provided at T2					
MOM, *n* (%)	91	71 (78.0)	92	81 (88.0)	0.071
DHM, *n* (%)	91	12 (13.2)	92	7 (7.6)	0.216
Mixed milk, *n* (%)	91	8 (8.8)	92	4 (4.3)	0.225

*DHM* donor human milk, *HC* head circumference, *MOM* mother’s own milk, *SD* standard deviation.

### Associations between enteral volume and protein intakes and hormone concentrations

Data on enteral volume intake were missing in the medical records for more than 50% of infants at T0 (before start of fortification), thus associations between daily enteral volume/protein intake and hormone concentrations were only analyzed at T1 and T2, combining data from the two fortification groups. At T1, the daily enteral volume intake (mL/kg/day) was positively associated with concentrations of insulin and C-peptide (both *P* < 0.001, Supplementary Table 1), corresponding to a 1.5% increase in insulin and a 0.9% increase in C-peptide per mL increase in total milk volume intake. At T2, the daily enteral volume intake was positively associated with concentrations of GIP, glucagon, and C-peptide (all *P* < 0.05, Supplementary Table 1). The estimates correspond to a 0.6% increase in GIP, a 0.4% increase in glucagon, and a 0.4% increase in C-peptide per mL increase in total milk volume intake. At T1, the total daily protein intake (g/kg/day) was significantly positively associated with concentrations of IGF-1 and C-peptide (all *P* < 0.05, Supplementary Table 2). The estimates correspond to a 7.3% increase in IGF-1 and a 17.9% increase in C-peptide per gram increase in total protein intake. At T2, the total daily protein intake associated positively with concentrations of GIP, C-peptide and leptin (all *P* < 0.05, Supplementary Table 2). The estimates correspond to a 17.7% increase in GIP, a 15.7% increase in C-peptide, and a 24.5% increase in leptin per gram increase in total protein intake. The type of fortification had no consistent effects on the milk volume and protein intake associations with hormone concentrations.

### Association between growth indices and hormone concentrations

Baseline characteristics of the infants with measurements of IGF-1 and other hormones are shown in Table 1. At T0 (postnatal age ~one week), leptin concentrations were negatively associated with weight ΔZ-scores from birth to 35 weeks PMA, and at T1 (postnatal age ~two weeks) IGF-1 concentrations were positively associated with weight and length ΔZ-scores from birth to 35 weeks PMA (Table 3 and Supplementary Table 3). Further, IGF-1 concentrations at T2 (postnatal age ~three weeks) were positively associated with weight ΔZ-scores (Table 3). SGA status did not significantly interact with the effects of IGF-1 on weight and length growth at any time point. No significant associations were found between hormone concentrations and ΔZ-scores for HC at any time point (Supplementary Table 4).

**Table 3:** Associations between plasma hormone concentrations and ΔZ-score for body weight from birth to 34 + 6 weeks postmenstrual age.

	Estimate	95% CI	Partial R^2^	*P*-value
T0				
IGF-1, ng/mL	0.003	[−0.010; 0.016]	0.002	0.663
GLP-1, pg/mL	–0.001	[−0.009; 0.008]	0.000	0.857
C-peptide, pg/mL	1.10^−5^	[−1.10^−4^; 1.10^−4^]	0.000	0.862
GIP, pg/mL	–0.001	[−0.004; 0.003]	0.003	0.637
Glucagon, pg/mL	4.10^−4^	[−0.002; 0.003]	0.001	0.733
Insulin, µIU/mL	0.005	[−0.005; 0.014]	0.011	0.321
Leptin, pg/mL	–0.002	[−0.004; -0.001]	0.093	0.004
PP, pg/mL	3.10^−4^	[−2.10^−4^; 0.001]	0.018	0.205
Glucose, mg/dL	–0.001	[−0.007; 0.006]	0.001	0.863
T1				
IGF-1, ng/mL	0.017	[0.006; 0.027]	0.095	0.002
GLP-1, pg/mL	–0.002	[−0.006; 0.003]	0.007	0.422
C-peptide, pg/mL	6.10^−5^	[−6.10^−5^; 2.10^−4^]	0.010	0.348
GIP, pg/mL	–4.10^−5^	[−0.002; 0.002]	0.000	0.967
Glucagon, pg/mL	–4.10^−4^	[−0.002; 0.002]	0.002	0.675
Insulin, µIU/mL	1.10^−4^	[−0.006; 0.006]	0.000	0.963
Leptin, pg/mL	–0.001	[−0.001; 6.10^−5^]	0.036	0.077
PP, pg/mL	3.10^−4^	[−1.10^−4^; 0.001]	0.024	0.150
Glucose, mg/dL	0.003	[−0.003; 0.008]	0.023	0.334
T2				
IGF-1, ng/mL	0.010	[0.001; 0.018]	0.053	0.024
GLP-1, pg/mL	2.10^−6^	[−0.007: 0.007]	0.000	0.999
C-peptide, pg/mL	9.10^−5^	[−2.10^−5^; 2.10^−4^]	0.028	0.119
GIP, pg/mL	0.001	[−0.001; 0.002]	0.013	0.281
Glucagon, pg/mL	–4.10^−4^	[−0.003; 0.003]	0.001	0.805
Insulin, µIU/mL	0.002	[−0.004; 0.008]	0.005	0.531
Leptin, pg/mL	–1.10^−4^	[−0.001; 0.001]	0.001	0.723
PP, pg/mL	–4.10^−5^	[−0.001; 4.10^−4^]	0.000	0.885
Glucose, mg/dL	0.002	[−0.001; 0.009]	0.005	0.648

Analyses include only infants with plasma samples available at T0 (postnatal age 7 ± 3 days), T1 (postnatal age 14 ± 3 days) and T2 (postnatal age 21 ± 3 days). IGF-1: *n* = 99, other hormones: *n* = 92, glucose: *n* = 46. The models are adjusted for gestational age at birth, small for gestational age and intervention. CI, 95% confidence interval; GIP, gastric inhibitory polypeptide; GLP-1, glucagon like peptide-1; IGF-1, insulin-like growth factor-1; PP, pancreatic polypeptide.

*CI* 95% confidence interval, *GIP* gastric inhibitory polypeptide, *GLP-1* glucagon like peptide-1, *IGF-1* insulin-like growth factor-1, *PP* pancreatic polypeptide.

### Growth and hormone concentrations in AGA and SGA infants over time

**Fig. 2:**
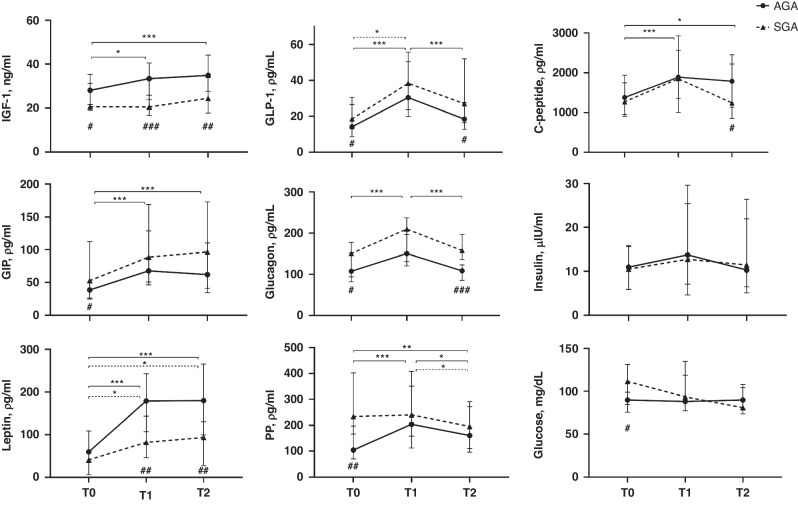
Temporal changes in plasma concentrations of hormones in very preterm infants born appropriate for gestational age (AGA) and small for gestational age (SGA), including only infants with plasma samples available at T0 (postnatal age 7 ± 3 days), T1 (postnatal age 14 ± 3 days) and T2 (postnatal age 21 ± 3 days). Plasma was analyzed for IGF-1 (*n* = 70 AGA versus 31 SGA), other hormones (*n* = 76 AGA versus 18 SGA), and glucose (*n* = 31 AGA versus 15 SGA). The models were adjusted for gestational age at birth and intervention. IGF-1 insulin-like growth factor-1, GLP-1, glucagon like peptide-1, GIP gastric inhibitory polypeptide, PP pancreatic polypeptide. Data is presented as median with interquartile range. *P < 0.05, **P < 0.01, ***P < 0.001 in the repeated measurement analysis (e.g. changes over time/age). ^#^*P* < 0.05, ^##^*P* < 0.01, ^###^*P* < 0.001 between SGA and AGA infants at each specific time.

Infants born SGA had a higher weight ΔZ-score (0.21 ± 0.61, *n* = 52 versus −0.33 ± 0.70, *n* = 171, mean ± SD, *P* < 0.001) and length ΔZ-score (0.11 ± 1.27, *n* = 38 versus −0.47 ± 1.29, *n* = 144, mean ± SD, *P* = 0.014) from birth to 35 weeks PMA than infants born AGA. No significant differences in ΔZ-score for HC were noted between SGA and AGA infants. In AGA infants, IGF-1, GLP-1, C-peptide, GIP, glucagon, leptin, and PP concentrations increased significantly between T0 and T1 (postnatal age ~one and two weeks, Fig. 2). Between T1 and T2 (postnatal age ~two and three weeks), there were significant decreases in GLP-1, glucagon, and PP concentrations in AGA infants, while no significant changes were observed in IGF-1, C-peptide, GIP, insulin, and leptin concentrations. In AGA infants, IGF-1, C-peptide, GIP, leptin, and PP concentrations increased between T0 and T2 (postnatal age ~one and three weeks), while no significant changes were noted in GLP-1, glucagon, and insulin concentrations. Glucose concentrations did not change between any of the time points. The age-related changes in hormone concentrations in SGA infants generally followed the pattern of the AGA infants. IGF-1 was an exception, since the concentrations remained stable, below 25 ng/mL, in the SGA infants and were significantly lower than in AGA infants at all three time points (Fig. 2). The leptin concentrations in SGA infants were lower at T1 and T2, and C-peptide concentrations lower at T2, than in AGA infants (Fig. 2). At T0, GIP, glucagon, PP, and glucose concentrations were significantly higher in SGA than in AGA infants. Also, SGA infants had higher concentrations of GLP-1 and glucagon at T2 (Fig. 2). No differences in insulin concentration, and QUICKI and HOMA2 indices (data not shown) were observed between AGA and SGA infants at any time point. The type of fortification had no consistent effects on the SGA status associations with hormone concentrations.

## Discussion

Considering the impaired growth and disturbances in glucose regulation in the first weeks after preterm birth,^27–29^ it is important to identify the associations between growth parameters, glucose-regulatory hormones and nutrient fortification at this critical time. Poor growth rates are commonly observed in VPIs,^48,49^ interacting with nutrition,^49–51^ metabolic-endocrine disruptions,^52^ immature organ functions and inflammatory mediators,^53^ but the underlying mechanisms are still unclear. Adequate nutrition and weight gain in early life are important for later neurodevelopment^1–5,54^ and catch-up growth.^55,56^ On the other hand, rapid early growth may predispose to metabolic programming of later cardiovascular diseases,^57,58^ insulin resistance^59^ and obesity.^60^ More insight is required into the factors associated with the type and amount of nutrient intake during the first weeks of life, when immature digestive and metabolic capacities restrict total nutrient intake and utilization.

In this study, all infants had reached full enteral feeding by the start of fortification (median postnatal day 8–9). After about 11 days, most infants were clinically stable and had regained their birth weight,^41^ making the first 1–3 weeks after preterm birth a highly relevant period to assess hormone levels and early biomarkers of growth. Fortification with the novel BC fortifier was associated with higher plasma GIP, GLP-1, glucagon and leptin concentrations. This may be explained by differences in the protein composition of the fortifiers (e.g. intact proteins like immunoglobulins, lactoferrin, growth factors and casein in BC versus hydrolyzed bovine milk whey proteins in CF^6,12–15,41^). In adults, whole protein versus hydrolyzed protein did not affect GIP concentrations^61^ but could induce a prolonged secretion of some hormones, including glucagon.^62^ The bioactive proteins in the BC fortifier may have indirect effects on endocrine hormone production by enhancing intestinal mucosal protection and growth, interacting with nutrient absorption and hormone release.^23–26^ Previous studies on BC products, and tests of these in piglets and cells, showed that BC bioactivity remains even after processing by gentle spray-drying, low temperature pasteurization (63 °C, 30 min) and gamma irradiation.^11,63,64^ We are currently investigating the effects of BC fortification on the fecal microbiota composition, but the effects seem to be small (unpublished data).

A slightly higher intake of human milk in the BC group at T1-T2, to attain similar growth rates between groups (Table 2), may also play a role. Also, although the differences did not reach statistical significance, the BC group received 0.2–0.3 g/kg/d more protein than the CF group at T1-T2. As previously discussed,^41^ reduced digestibility of BC proteins and a slightly lower proportion of MOM feeding (which contains more protein than DHM) may explain why the feeding volume was adjusted to higher levels in BC than CF infants to achieve target weight gains. Incretins are secreted from enteroendocrine cells in response to enteral milk intake and influence the production of hormones from the endocrine pancreas.^37–40^ Daily enteral volume and total protein intake were positively associated with several glucose-regulatory hormones. One mL increase in daily intake of fortified human milk (with MOM being the primary milk type provided in ~82–83% of all the infants, Table 2) increased the concentrations of C-peptide, GIP, insulin and glucagon by ~0.4–1.5%, while one gram increase in protein intake increased the C-peptide, GIP, IGF-1 and leptin concentrations by ~7–24%, at T1 and T2. Yet, adjustment for enteral milk/protein intake (or baseline values) had little impact on the general results regarding the BC fortification effects, and we conclude that both the type of fortifier, milk volume and protein intake affect the levels of glucose-regulatory hormones in preterm infants. With >80% of the included infants receiving primarily MOM, and only ~10% receiving primarily DHM, it was not meaningful to study the effects of milk type on hormone levels in this study. Further studies are required to delineate the cause-effect relationships and how factors other than dietary protein (e.g. fat, carbohydrates, vitamins, minerals) may affect hormone levels and growth.

The potential physiological effects of the BC-induced increments (~20–30%) in some glucose-regulatory hormones are unknown. Glucagon controls hepatic release of glucose, yet blood glucose levels were similar between the groups, and BC fortification did not influence insulin sensitivity or resistance (QUICKI/HOMA indices). Possibly, concomitant increases in GLP-1 and GIP affected gut motility and food passage and counteracted plasma glucose increase. The glucose-regulatory effects of BC fortification were subtle, but potential intestinotrophic effects of BC warrant further investigation.

In the past decades, only few attempts have been made to correct endocrine disruptions and regulators of growth and metabolism in VPIs. This is partly due to concerns regarding safety, efficacy and mechanisms of hormonal interventions. While administration of prenatal glucocorticoids and postnatal insulin support lung maturation^65^ and glucose tolerance,^29^ respectively, no long-term hormonal therapies have been tested to combat poor growth in neonatology. Low circulating IGF-1 levels are associated with reduced growth^17–21^ and several perinatal morbidities,^66–69^ and an international trial is ongoing to verify if supplemental IGF-1, combined with its key binding protein BP-3, prevents morbidities in extremely preterm infants (clinicaltrials.gov registration: NCT03253263). The current study found that IGF-1 concentrations at T1 and T2 (two to three weeks of age) were positively associated with ΔZ-scores for weight and length. The estimates suggest that the weight ΔZ-score increases by 0.01–0.02 for every ng increase in IGF-1. Given that the weight ΔZ-score was ~0.2 in the cohort, our study underlines the importance of IGF-1 in postnatal growth. Yet, IGF-1 explained only ~5–10% of the overall variation in growth rates in partial correlation analyses, suggesting that factors other than IGF-1 affect body weight growth. The majority of circulating IGF-1 is produced by the liver. In neonates, hepatic IGF-1 production is stimulated by insulin, and indirectly by nutrient input, whereas glucocorticoids inhibit IGF-1 release.^22,52^

In contrast to IGF-1, leptin concentrations at T0 (one week of age) were negatively associated with ΔZ-scores. In adults, leptin is a satiety-regulating hormone that induces weight loss and reduces food intake and glucose and insulin levels.^70^ Leptin is secreted by adipocytes and is positively associated with body weight and body mass index (BMI) at birth and during the first week of life in preterm infants.^71,72^ The leptin concentrations in our VPI cohort were also markedly lower than previously reported in term infants after birth.^39^ Similar to our study, Ong et al.^73^ found that cord leptin levels in term infants correlated negatively with weight gain between birth and four months of age. Also, term-born infants showing catch-up growth during the first year of life had markedly lower cord leptin levels than infants with no catch-up growth.

By combining the data from the two fortification groups, we assessed the temporal changes in glucose-regulatory hormones with advancing postnatal age in VPIs born AGA or SGA. Plasma concentrations of all the studied hormones increased from T0 to T1 (one to two weeks of age) in AGA infants (for insulin, *P* = 0.06). Concentrations of IGF-1, C-peptide, GIP, insulin and leptin were stable between T1 and T2, whereas GLP-1, glucagon and PP concentrations peaked at T1 and decreased at T2. Previous studies have described a similar peak in GLP-1 and PP concentrations at weeks 2-3 after preterm birth,^37,39,74^ maybe due to a combination of age- and fortification-induced increases in hormone secretion, combined with low clearance during the first weeks of life (e.g. immaturity of kidneys, liver and circulating peptidases). Frequent enteral feedings and low clearance may also explain that some basal hormone concentrations (e.g. insulin, GLP-1, GIP, glucagon at T1-T2) were generally higher in our VPIs, than in healthy adults, despite similar basal glucose concentrations.^75,76^ Also, some incretins and satiety factors (GLP-1, GIP, PP) were higher in SGA versus AGA infants, which, together with increased glucose concentrations in SGA infants at T0, indicates enhanced feeding responses in this subgroup of infants, or reduced clearance with disrupted regulation and feedback mechanisms.

In general, the temporal changes in hormone levels in SGA infants showed the same trends as in AGA infants, except for IGF-1, which remained low in SGA infants, like in previous studies.^20^ While IGF-1 concentrations were lower in SGA infants, weight and length growth were higher than in AGA infants, suggesting that circulating IGF-1 might have different effects on growth depending on the metabolic and developmental status of the infant. SGA infants are born with very small fat deposits and loose less weight just after birth, making their body weight ΔZ-scores high compared with AGA infants. However, in this study we did not find the IGF-1 effects on weight growth to be significantly dependent on SGA status.

Another study on very low birth weight infants (Zamir et al.^77^ mean GA 27.2 weeks, *n* = 48) reported higher mean insulin and glucose concentrations than in our study, while C-peptide concentrations were similar, using similar methods of analyses. Accordingly, leptin concentrations and HOMA2 index were higher, while QUICKI index was lower. This suggests that the infants in our study were more insulin sensitive, perhaps due to their slightly higher mean GA at birth.^27^ In another study on preterm infants (Salis et al.^78^ PMA ≤ 32 weeks, *n* = 113–138), insulin and glucose concentrations were similar to our cohort, while C-peptide concentrations were lower. However, methodical (assays, timing) and nutritional (parenteral versus enteral nutrition, DHM, MOM, formula) differences make comparison of results between studies difficult.

Our study has several limitations. Importantly, we cannot separate the potential effects of increasing postnatal age from increasing amounts of fortifiers or volumes of human milk with advancing age. The protocol followed a pragmatic study design where limited volumes of sampled blood were allowed, which led to differences in the number of samples included at different time points, and specifically the sample sizes for glucose measurements (and thus QUICKI and HOMA indices) ended up being smaller than desirable. The intention was to take blood samples just before the next meal since feeding stimulates many incretins, glucose and insulin. However, some variation in sample time is unavoidable and this may have contributed to inter- and intra-individual variation. We generally found lower concentrations of incretins, particularly GLP-1, compared with those in other studies of preterm infants.^37,39,77^ We did not add protease inhibitors to our collection tubes and we cannot exclude that this affected the level of relatively short-lived peptides, such as glucagon, GLP-1 and GIP. Only a small part of the overall variation in growth rates was explained by the studied hormone concentrations, suggesting that the hormone levels are not critical determining factors for early postnatal growth. Moreover, the transient age-, meal- and feeding-related changes in glucose-regulating hormone concentrations during the first postnatal weeks make these hormone levels challenging to use as biomarkers of growth or other clinical outcomes.

In conclusion, our study helps to understand the variation in glucose-regulatory hormones in the early life of VPIs. The clinical relevance of the BC-induced hormone increments is uncertain, but our results support that IGF-1 plays a role in early postnatal growth in VPIs. Still IGF-1 levels explained only a small part of the overall variation in growth rates. Other glucose-regulatory hormones did not associate positively with growth rate, were highly variable and modified by AGA/SGA status, type of fortifier, enteral milk intake, and advancing postnatal age. Overall, the studied glucose-regulatory hormones do not seem to be appropriate biomarkers of early postnatal growth for individual VPIs. Additional well-controlled studies in preterm infants or animal models are required to elucidate the complex relationships between different metabolic markers (hormones, metabolites), nutrition and early postnatal growth.

## Supplementary information

Supplementary figure 1

Supplementary table 1

Supplementary table 2

Supplementary table 3

Supplementary table 4

## Data Availability

Data described in the manuscript, code book, and analytic code will be made available upon request pending application and approval by Danish law. The study was approved by the ethical committee of the Region of Southern Denmark (S-20130010, S-20170095) and the Danish Data Protection Agency (17/33672). All data were entered into the online database, REDCap in OPEN, in the Region of Southern Denmark.
