# Supplementary figures and images for: Glucose-regulatory hormones and growth in very preterm infants fed fortified human milk
Source: Pediatr Res. 2024 Apr 5;96(3):713–22. doi: 10.1038/s41390-024-03166-8 (PMC11499248; doi:10.1038/s41390-024-03166-8)

**Supplementary figure 1.** Participant flow chart.

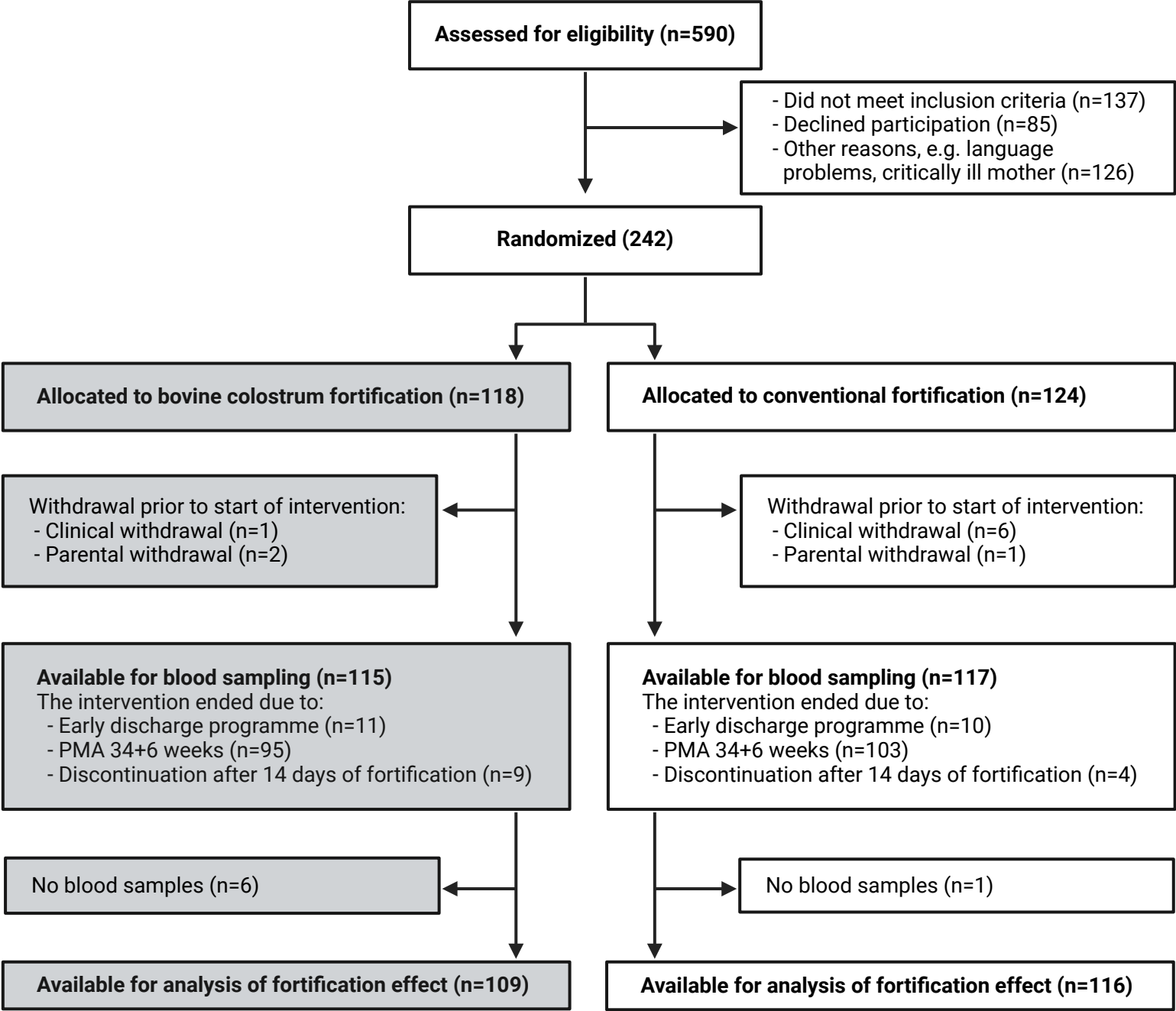

Supplement: Supplementary file 1 — Supplementary figure 1 [file 41390_2024_3166_MOESM1_ESM.pdf]
